# Predictors of Emergency Surgery in Acute Ischaemic Colitis: A Systematic Review and Meta-Analysis

**DOI:** 10.3390/jcm15218539

**Published:** 2026-11-02

**Authors:** Naomi Abara, Abdulazeez Mustapha, Mohamed Elsaigh, Awofaa Gogo-Abite

**Affiliations:** 1 Department of General Surgery, Royal Cornwall Hospital NHS Trust, Truro TR1 3LJ, UK; m.elsaigh@nhs.net (M.E.); awofaa.gogo-abite1@nhs.net (A.G.-A.); 2 Department of General Surgery, Chelsea and Westminster Hospital NHS Foundation Trust, London SW10 9NH, UK; abdulazeez.mustapha1@nhs.net

**Keywords:** ischaemic colitis, emergency surgery, colectomy, risk factors, predictors, prognostic factors

## Abstract

**Background:** Acute ischaemic colitis ranges from reversible mucosal injury to transmural necrosis requiring emergency resection. This review aimed to identify factors associated with emergency surgery, quantify comparable unadjusted associations, and assess their robustness. **Methods:** PubMed, Cochrane Library, Embase, and Ovid MEDLINE were searched to 10 June 2026. Adult cohort studies reporting predictors or outcomes relevant to emergency surgery for ischaemic colitis (IC) were included. Surgery-specific unadjusted odds ratios (ORs) were pooled only when at least three independent, clinically comparable studies were available, using random-effects models with restricted maximum-likelihood estimation; Hartung–Knapp confidence intervals were used for primary inference. Risk of bias was assessed with QUIPS, and certainty with GRADE adapted for prognostic-factor evidence. The protocol was registered in PROSPERO (CRD420261411575). **Results:** Twenty-six cohort studies were included; five contributed data to at least one of three retained meta-analyses. Conventional REML estimates suggested higher odds of emergency surgery with hypotension (OR 3.53, 95% CI 1.10–11.28; I^2^ = 75.0%) and right-colonic involvement (OR 4.60, 95% CI 1.47–14.40; I^2^ = 80.4%), while fever was imprecise (OR 3.42, 95% CI 0.59–19.79; I^2^ = 86.3%). Hartung–Knapp 95% CIs included one for hypotension (0.30–41.71), fever (0.08–142.15), and right-colonic involvement (0.66–31.86), and no pooled association remained statistically robust. GRADE certainty was very low for all three. **Conclusions:** Hypotension and right-sided disease remain clinically plausible candidate risk markers, but the evidence is sparse and very uncertain and does not establish independent predictors or operative thresholds. Prospective multicentre studies using standardised definitions are needed to develop and externally validate a surgery-specific prediction model.

## 1. Introduction

Ischaemic colitis (IC) is the most common form of gastrointestinal ischaemia requiring emergency hospital admission, encompassing a spectrum from transient mucosal hypoperfusion to transmural necrosis of the colon [1,2,3]. Management commonly involves the surgical team with gastroenterology input, although practice varies across centres [3]. It is a disease of the elderly, occurring in occlusive and non-occlusive vascular disease [4], with the latter being the most common and the focus of this review. The incidence of ischaemic colitis in general populations ranges from 4.5 to 44 cases per 100,000 person-years [5]. In a US population-based cohort, the incidence increased from 6.1 to 22.9 cases per 100,000 person-years between 1976–1980 and 2005–2009 [5,6]. The management is largely conservative, with bowel rest, fluid resuscitation and treatment of precipitating factors; however, a small number deteriorate or present with irreversible colonic ischaemia and require emergency colonic resection [3].

A recent study estimated that approximately 15% of patients undergo surgical treatment, while emphasising the limited reporting of surgical outcomes [7]. When surgery is required, it is generally performed in patients with advanced physiological deterioration, peritonitis, gangrene or perforation and is associated with substantial postoperative morbidity and mortality [2,3,8,9]. Early recognition of patients at increased risk of emergency surgery could therefore facilitate closer monitoring, early senior surgical involvement and timely escalation of care.

Clinical, biochemical, anatomical, radiological and endoscopic features have all been proposed as markers of severe disease [2,3,10]. However, the reported associations have been inconsistent, frequently originate from small retrospective cohorts, and are sometimes based on composite outcomes combining surgery with death. These limitations have impeded the development of reliable surgery-specific risk-stratification tools and created a need for a systematic synthesis focused specifically on emergency operative intervention in index admission. Although clinically informative, composite outcomes do not establish which factors specifically identify patients who progress to emergency surgery.

This systematic review and meta-analysis therefore aimed to identify factors associated with emergency surgery during an acute episode of IC, quantify sufficiently comparable unadjusted associations, and test their robustness to predictor-definition restrictions and small-sample confidence-interval methods.

## 2. Methods

This systematic review and meta-analysis are reported in accordance with the Preferred Reporting Items for Systematic Reviews and Meta-Analyses (PRISMA) 2020 statement [11]. The study protocol was registered in the International Prospective Register of Systematic Reviews (registration number CRD420261411575). The completed PRISMA 2020 checklist is provided in Supplementary Table S1.

### 2.1. Search Strategy

A comprehensive literature search was performed by two investigators (NA and MA) on 10 June 2026 across PubMed, Cochrane Library, Embase and Ovid MEDLINE. Additionally, the reference lists of included studies and relevant reviews were manually screened to identify any additional eligible studies. The core search terms included ischaemic/ischemic colitis, emergency surgery or colectomy, and prediction, risk or prognosis. Supplementary Table S2 provides comprehensive details of the search strategy.

### 2.2. Study Selection, Eligibility Criteria and Screening

Only publications in English and studies involving humans were considered. All original research articles included had to demonstrate an association between preoperative demographic, comorbidity, clinical, laboratory, endoscopic, or imaging factors and the need for emergency surgery, progression to severe disease requiring surgery, or outcomes following emergency surgery for acute ischaemic colitis in adults. For inclusion in the primary meta-analysis, studies were required to provide a surgery-specific comparison and either an unadjusted odds ratio (OR) with a 95% confidence interval (CI) or sufficient data to construct a 2 × 2 table. Predictors were pooled only when reported in at least three independent, clinically comparable studies.

Non-English reports, reviews, editorials or comments, reports without predictor assessment, reports without a surgical outcome, and studies confined to postoperative colonic ischaemia following vascular surgery were excluded. Studies with composite surgery/death outcomes, mortality-only analyses or cohorts in which every participant underwent colectomy remained eligible for the qualitative review but were not treated as direct evidence of progression to surgery. Composite severe-outcome and postoperative studies were retained as contextual evidence to assess whether predictors associated with emergency surgery also recurred across broader adverse outcomes and postoperative mortality.

In accordance with the eligibility criteria, the articles included were analysed independently by two reviewers (NA and ME), and the proportion of agreement was 95%. Discrepancies were resolved through discussion between the two authors until a consensus was reached. Further disagreements were resolved with a third reviewer (MA). Item-level screening decisions were not retained in a form that permitted reconstruction of Cohen’s κ; no κ value was retrospectively estimated.

### 2.3. Data Extraction

Two reviewers (NA and MA) independently extracted data from the included studies using a prespecified standardised data-extraction form. Extracted information included the first author, year of publication, country, study design and data source, study period, sample size, participant characteristics, definitions of ischaemic colitis and emergency surgery, predictors assessed, and reported effect estimates with corresponding 95% confidence intervals. Where available, raw event data were extracted to construct 2 × 2 contingency tables and calculate unadjusted odds ratios. Disagreements were resolved through discussion and consensus, with adjudication by a third reviewer when required.

### 2.4. Outcome Definition and Predictor Harmonisation

The primary outcome was the requirement for operative intervention during the index acute presentation or hospital admission for ischaemic colitis, including urgent laparotomy, colectomy or colonic resection. Owing to variation in the terminology used across studies, these encompassed procedures described as emergency or immediate surgery, surgery during the index admission, in-hospital colectomy, and surgery following clinical deterioration or failure of initial non-operative management. Postoperative mortality and complications were considered distinct outcomes and were not pooled with the requirement for surgery. Elective surgery for late complications was not included in the primary quantitative synthesis.

Predictors were harmonised only when their underlying clinical construct, direction of effect and reference category were sufficiently comparable. Effect estimates were consistently coded such that an odds ratio greater than 1 indicated increased odds of emergency surgery. Continuous and categorical representations of the same predictor were not combined, and adjusted estimates were not pooled with unadjusted estimates. Clinically distinct definitions were retained as separate predictors or evaluated using definition-restricted sensitivity analyses.

### 2.5. Risk of Bias and Certainty of Evidence

Risk of bias was assessed independently by two reviewers (NA and MA) using the Quality in Prognosis Studies (QUIPS) tool, covering study participation, attrition, prognostic-factor measurement, outcome measurement, study confounding, and statistical analysis/reporting [12]. Disagreements were resolved by discussion and consensus. Domain-level judgements were rated as low, moderate, or high risk; no overall numerical score or overall QUIPS judgement was calculated (Supplementary Table S3). Certainty of evidence for the three retained pooled predictors was assessed using GRADE adapted for prognostic-factor research [13], considering risk of bias, inconsistency, indirectness, imprecision, and publication bias (Supplementary Table S5).

### 2.6. Synthesis and Statistical Analysis

Meta-analysis was performed for each predictor when at least three independent studies reported sufficiently comparable unadjusted surgery-specific data. Odds ratios (ORs) and 95% confidence intervals (CIs) were extracted directly or calculated from reported 2 × 2 data. Where a 2 × 2 table contained a zero cell, a continuity correction of 0.5 was added to all four cells for that study-level estimate.

Pooled ORs and 95% CIs were calculated using a random-effects meta-analysis, with between-study variance estimated using restricted maximum likelihood (REML) [14,15]. Statistical heterogeneity was assessed using Cochran’s Q test, I^2^ and τ^2^. An I^2^ value > 50% or *p* ≤ 0.10 indicated substantial heterogeneity. Predictors unsuitable for pooling and adjusted estimates were summarised narratively. Funnel plots were not produced because fewer than 10 studies contributed to each analysis.

Hartung–Knapp 95% CIs were used as the primary inferential intervals for the retained random-effects meta-analyses, with conventional normal-approximation REML CIs shown for comparison [16]. Definition-restricted analyses were retained as sensitivity analyses; where only two studies remained, these were treated as exploratory stress tests and interpreted cautiously. All analyses were conducted in R version 4.6.1 using the meta package version 8.5.0. Protocol deviations from the PROSPERO registration included increasing the minimum pooling threshold from two to three independent clinically comparable studies, adding Hartung–Knapp and definition-restricted analyses, QUIPS risk of bias assessment, and adding prognostic-factor GRADE.

## 3. Results

### 3.1. Study Selection

**Figure 1:**
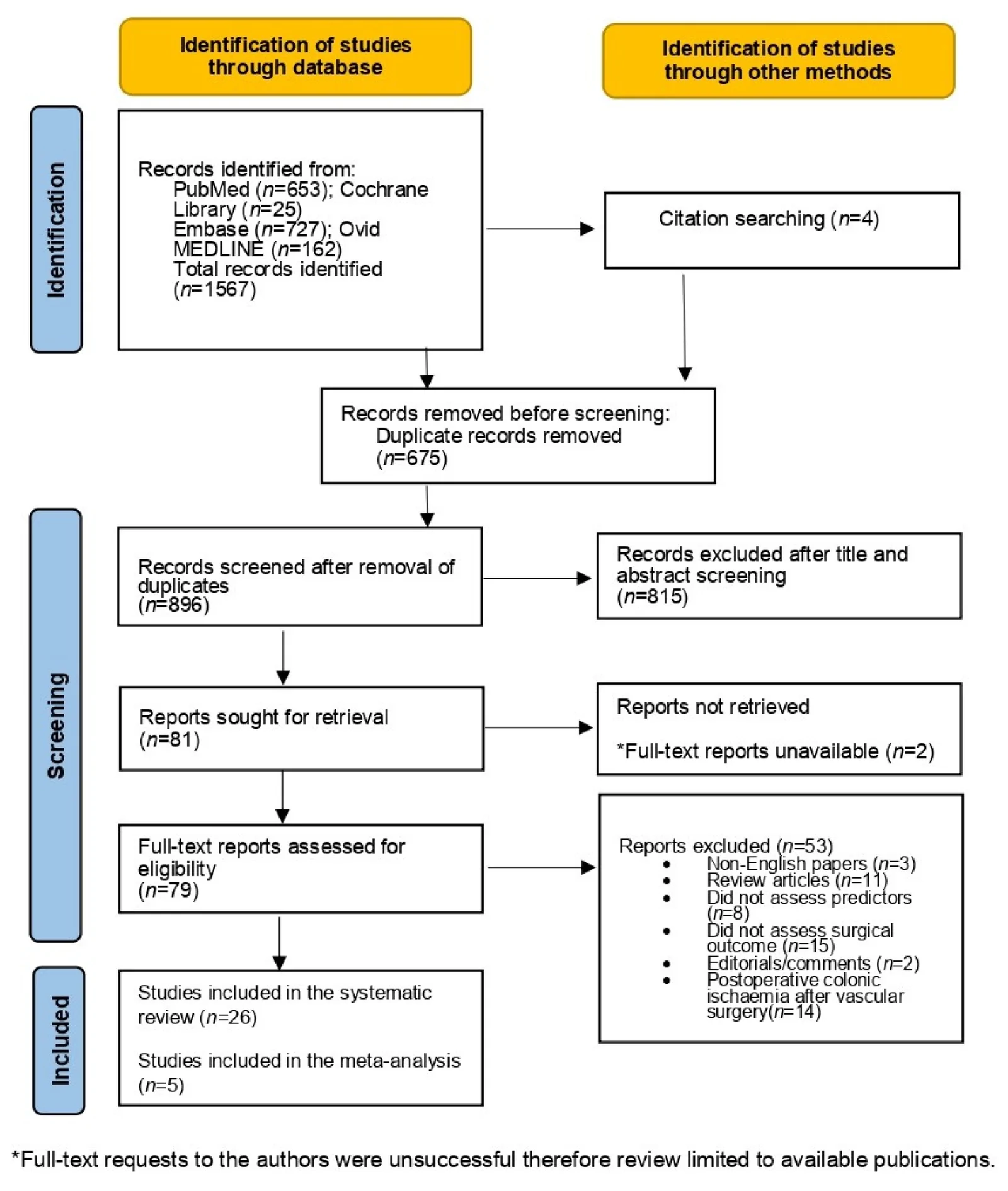
PRISMA 2020 flow diagram for systematic reviews incorporating database, registers and citation searches [11]. PRISMA: Preferred Reporting Items for Systematic Reviews and Meta-Analyses.

The database searches identified 1567 records (PubMed 653, Cochrane Library 25, Embase 727 and Ovid MEDLINE 162), and citation searching identified four additional records. After removal of 675 duplicates, 896 records underwent title and abstract screening and 815 were excluded. Eighty-one reports were sought; two full texts could not be obtained. Of 79 full-text reports assessed, 53 were excluded: non-English publication (*n* = 3), review article (*n* = 11), no predictor assessment (*n* = 8), no assessment of surgery (*n*= 15), editorial/comment (*n* = 2), or postoperative colonic ischaemia following vascular surgery (*n* = 14). Twenty-six studies shown in Figure 1 were included in the systematic review, and five studies with sufficient data contributed to the meta-analysis.

### 3.2. Study Characteristics

All included cohort studies were published between 1992 and 2024 [1,8,9,17,18,19,20,21,22,23,24,25,26,27,28,29,30,31,32,33,34,35,36,37,38,39]. Twenty-three (88.5%) were retrospective, and three (11.5%) prospective. Thirteen studies were conducted in Europe, eight in North America, four in Asia and one in the Middle East. Twenty-four used clinical or medical-record cohorts, and two used administrative claims databases. The median primary IC cohort was 123 patients (sample size range 32–106,705). Eleven reports evaluated a direct surgery or colectomy outcome, ten used a composite severe outcome that included surgery and/or death, and five studied postoperative outcomes in surgical cohorts. Table 1 summarises the included studies and results.

Five studies—Paterno et al., Kim et al., Brandt et al., Medina et al. and Scharff et al.—contributed compatible unadjusted surgery-specific data to at least one of the three predictor meta-analyses [21,23,33,35,39]. The remaining evidence was synthesised narratively and presented as individual study estimates because outcomes, predictor definitions, or the number of comparable studies were insufficient for pooling. Three to four studies contributed to each retained pooled estimate. Overall, the included studies were characterised by important risk-of-bias concerns, with 23 of 26 studies rated at high risk in at least one QUIPS domain. High-risk judgements occurred most frequently in study confounding, statistical analysis/reporting, and prognostic-factor measurement (Supplementary Table S3).

### 3.3. Predictors of Surgery Included in the Meta-Analysis

**Figure 2:**
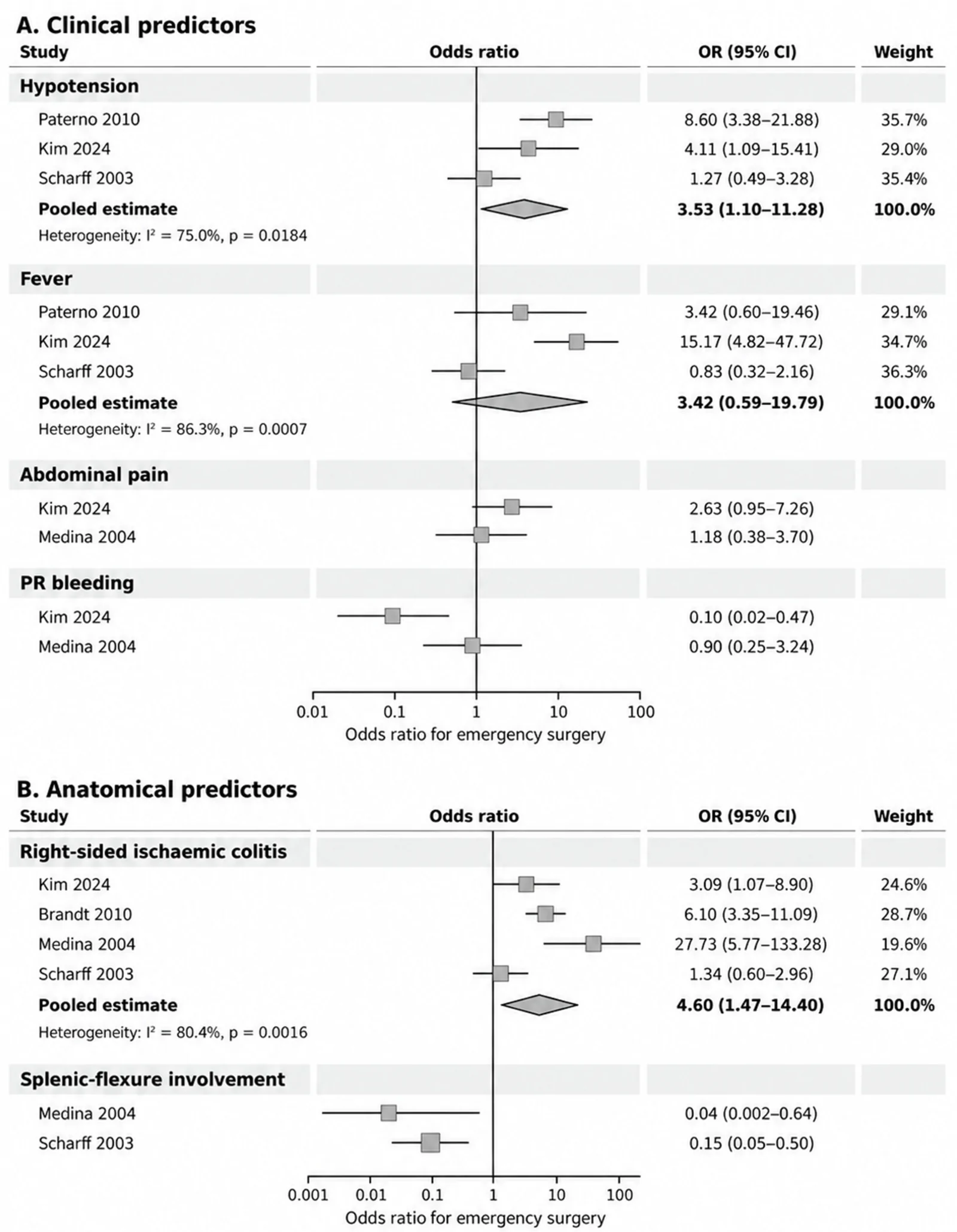
(**A**) Clinical predictors: hypotension, fever, abdominal pain and PR bleeding; (**B**) anatomical predictors: right-colonic involvement and splenic-flexure involvement. Squares represent study-specific odds ratios (ORs), with horizontal lines indicating 95% confidence intervals (CIs). Vertical reference line at OR = 1 represents no association between the predictor and emergency surgery. Diamond represents pooled random-effect estimates using restricted maximum-likelihood (REML) estimation and are shown only where at least three clinically comparable studies contributed. Abdominal pain, PR bleeding and splenic-flexure involvement were each reported by only two studies and are therefore presented as individual study estimates without pooled effects or heterogeneity statistics. Study weights are shown only for predictors included in pooled analyses. The pooled diamonds display conventional REML 95% CIs; Hartung–Knapp-adjusted CIs used for primary statistical inference are reported separately in the accompanying Hartung–Knapp results table. OR > 1 indicates greater odds of emergency surgery. Studies shown are Paterno et al. (2010) [21], Kim et al. (2024) [23], Brandt et al. (2010) [33], Medina et al. (2004) [35], and Scharff et al. (2003) [39].

Three predictors were included in the primary unadjusted meta-analysis. Among the clinical and anatomical predictors shown in Figure 2, hypotension was associated with greater odds of emergency surgery in the conventional REML analysis (three studies; OR 3.53, 95% CI 1.10–11.28; I^2^ = 75.0%; τ^2^ = 0.7595) [21,23,39]. Right-colonic involvement was also associated with greater odds of surgery (four studies; OR 4.60, 95% CI 1.47–14.40; I^2^ = 80.4%; τ^2^ = 1.0863) [23,33,35,39]. Both estimates showed substantial between-study heterogeneity.

Fever showed a directionally positive but imprecise association with emergency surgery (three studies; OR 3.42, 95% CI 0.59–19.79; I^2^ = 86.3%; τ^2^ = 1.9681) [21,23,39]. Hartung–Knapp 95% CIs included one for hypotension (0.30–41.71), fever (0.08–142.15), and right-colonic involvement (0.66–31.86), and no pooled association remained statistically robust (Table 2).

Abdominal pain, PR bleeding, and splenic-flexure involvement were each reported by only two studies and are therefore shown in Figure 2 as individual study estimates without pooled effects or heterogeneity statistics [23,35,39]. In Medina et al., splenic-flexure involvement contained no surgical events in the exposed group; a continuity correction of 0.5 was therefore applied to all four cells when calculating the study-level estimate [35].

**Figure 3:**
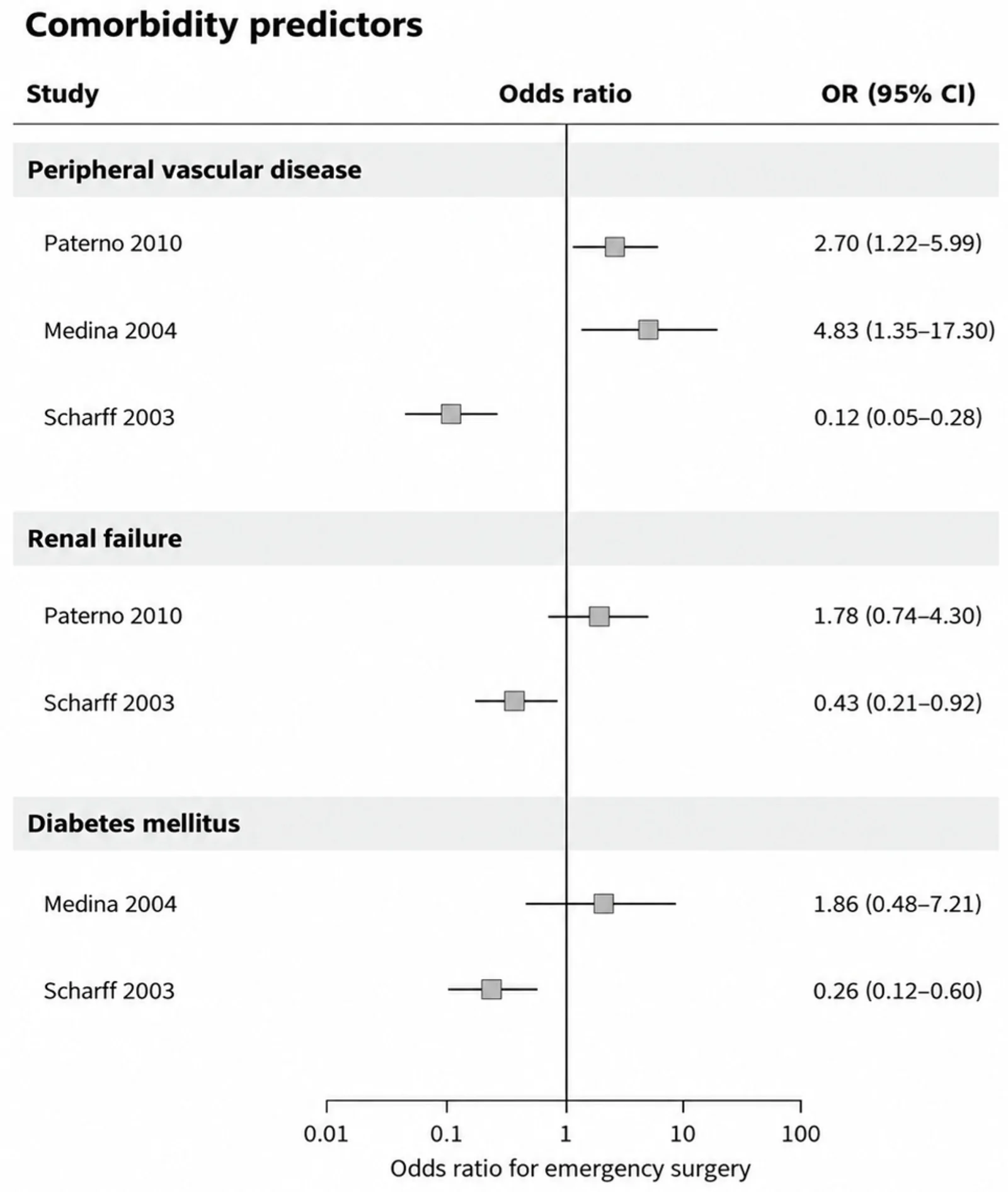
Comorbidity predictors of emergency surgery in ischaemic colitis. Squares represent study-specific odds ratios (ORs), with horizontal lines indicating 95% confidence intervals (CIs). Vertical reference line at OR = 1 represents no association between the predictor and emergency surgery. No pooled estimates are presented. Peripheral vascular disease was not pooled because the reported predictor definitions were not sufficiently comparable across studies, while renal failure and diabetes were each reported by only two studies. Accordingly, these predictors are presented descriptively as individual study estimates without pooled effects, study weights or heterogeneity statistics. OR > 1 indicates greater odds of emergency surgery. Studies shown are Paterno et al. (2010) [21], Medina et al. (2004) [35], and Scharff et al. (2003) [39].

Comorbidity predictors are shown in Figure 3. Peripheral vascular disease was not pooled because the reported predictor definitions were not sufficiently comparable across studies, while renal failure and diabetes mellitus were each reported by only two studies [21,35,39]. These predictors are therefore presented as individual study estimates without pooled effects. In Scharff et al., the study-level estimates were inverse for vascular disease (OR 0.12), renal failure (OR 0.43), and diabetes mellitus (OR 0.26); these findings reflect associations with observed operative treatment and should not be interpreted as protective effects.

### 3.4. Predictors of Surgery Not Included in the Meta-Analysis

Adjusted analyses and narrative synthesis identified additional surgery-specific associations, but these data were insufficiently comparable for pooling. In Paterno et al., free intraperitoneal fluid and absence of PR bleeding were independently associated with delayed surgery following initial conservative management [21]. In Kim et al., right-sided involvement, fever, and absence of rectal bleeding remained associated with progression to surgery after multivariable adjustment [23]. These adjusted findings were kept separate from the unadjusted meta-analysis because predictor definitions and covariate sets differed.

Beyond these surgery-specific analyses, studies using composite severe outcomes, mortality endpoints or emergency-surgical cohorts repeatedly reported adverse features, including haemodynamic instability or shock, tachycardia, peritoneal signs, absence of rectal bleeding, right-sided or extensive disease, renal dysfunction, inflammatory or metabolic disturbance, and adverse computed tomography (CT) or endoscopic findings [1,8,9,19,20,22,25,26,27,28,29,30,34,36,37,38,39].

Several reports developed or evaluated multivariable severity or mortality tools based on combinations of clinical, anatomical, radiological, endoscopic, or postoperative variables [25,27,30,37]. However, because their endpoints and intended populations differed, none represented an externally validated prediction model for emergency surgery that was applicable across clinical settings. The complete study-level evidence map, including adjusted, composite-outcome, and postoperative findings, is presented in Table 1.

### 3.5. Sensitivity Analyses

Definition-restricted sensitivity analyses were based on two studies each. Restricting hypotension to studies reporting systolic blood pressure <90 mmHg yielded OR 3.31 (95% CI 0.51–21.58; I^2^ = 87.4%); restricting fever yielded OR 1.39 (0.36–5.28; I^2^ = 48.9%); and restricting right-colonic involvement to the two closest anatomical definitions yielded OR 10.85 (2.57–45.86; I^2^ = 68.0%). These analyses were treated as exploratory stress tests rather than primary inference.

Hartung–Knapp CIs for the definition-restricted k = 2 analyses were extremely wide and all crossed one, reflecting the single degree of freedom available (Supplementary Table S4). GRADE certainty, shown in Supplementary Table S5, was very low for hypotension, fever, and right-colonic involvement because of serious or very serious concerns regarding risk of bias, inconsistency, indirectness, and imprecision; publication bias could not be assessed reliably with only three to four studies.

## 4. Discussion

Acute ischaemic colitis presents a challenging surgical decision. Most patients recover with supportive management, whereas a smaller group develop irreversible colonic injury or progressive physiological deterioration, requiring emergency resection [2,3,7]. The clinical dilemma is to recognise when continued conservative management is becoming unsafe without exposing patients with potentially reversible disease to unnecessary major surgery. This review identified 26 cohort studies addressing predictors or outcomes relevant to emergency surgery; 11 examined a direct surgery or colectomy outcome, and five contributed sufficiently comparable unadjusted data to at least one of three retained meta-analyses. To our knowledge, this is the first systematic review and meta-analysis to focus specifically on predictors of emergency surgery during index admission in patients with acute ischaemic colitis. The principal contribution of this review is its distinction of factors associated with emergency surgery from those associated with composite severity, mortality or postoperative prognosis.

Conventional REML analyses suggested higher odds of emergency surgery with hypotension and right-colonic involvement, while fever was imprecise. However, all Hartung–Knapp 95% CIs included the null value, and GRADE certainty was very low for each retained pooled predictor. Definition-restricted analyses also remained highly uncertain. The findings therefore identify clinically plausible candidate risk markers rather than established independent predictors or operative thresholds.

This distinction is particularly important because ischaemic colitis predominantly affects older adults, usually with existing cardiovascular, renal and other systemic comorbidities that limit physiological reserve [2,3,6]. Approximately 15% of patients undergo surgical treatment, and those requiring emergency colonic resection experience substantial postoperative morbidity and mortality [7,8,9,20,25,26]. It is therefore crucial to identify clinical deterioration early enough to allow operative and critical-care planning before further organ dysfunction reduces the patient’s operative tolerance.

Physiological findings are best interpreted within the context of disease progression. Hypotension may represent a low-flow state, contributing to colonic injury, impaired physiological compensation, or systemic deterioration secondary to advanced ischaemia. Circulatory compromise is recognised as a feature of severe disease in clinical guidance and previous reviews [2,3,10]. However, the included studies did not consistently distinguish transient hypotension at presentation from persistent hypotension despite resuscitation, limiting interpretation of its prognostic significance. Hypotension should therefore prompt urgent reassessment and escalation and be interpreted alongside response to resuscitation and organ dysfunction, rather than act as an isolated indication for colectomy. Similarly, the imprecise pooled finding for fever and sparse study-level evidence for renal dysfunction do not exclude their relevance when they occur as part of progressive systemic deterioration.

Anatomical distribution and clinical presentations may provide additional information. Right-sided involvement showed higher estimated odds of surgery, consistent with previous evidence linking right-colonic disease to severe outcomes [2,3,10,33,38]. Notably, as shown in Table 1, right colonic involvement recurred across surgery-specific, composite adverse-outcome and postoperative mortality studies, although differences in populations, definitions and outcome timing preclude direct comparison. This distribution may reflect more extensive low-flow injury and should raise suspicion of acute mesenteric ischaemia when the presentation is atypical or disproportionate [2,3]. However, definitions varied from isolated right-colon ischaemia to any right-colonic involvement, and substantial heterogeneity persisted. Right-sided disease should therefore lower the threshold for close monitoring and early senior surgical assessment but should not determine operative treatment in isolation.

Rectal bleeding also requires contextual interpretation. The two unadjusted surgery-specific study estimates were not pooled, whereas adjusted analyses by Paterno et al. and Kim et al. identified the absence of rectal bleeding as an adverse feature [21,23]. These findings are not directly comparable because predictor coding and analytical methods differed. Rectal bleeding is less frequent in right-sided IC [3] and should be interpreted alongside haemodynamic status, abdominal findings, disease extent, and response to treatment.

Splenic-flexure involvement was reported by only two studies, and both study-level estimates were below one, but the evidence was sparse, definition-dependent, and affected by a zero cell in one study, so it should not be interpreted as a protective finding.

The sparse and inconsistent study-level evidence for abdominal pain, peripheral vascular disease, renal failure, and diabetes should not be interpreted as evidence of no clinical relevance. In particular, the inverse comorbidity estimates reported by Scharff et al. should not be interpreted as evidence of a protective effect, as the study did not report whether operative fitness influenced treatment decisions [39].

The adjusted and narratively synthesised evidence in Table 1 provides further support for a multidimensional approach to risk assessment, although its clinical application remains limited. Paterno et al. identified free intraperitoneal fluid on computed tomography and the absence of rectal bleeding as factors associated with surgery after initial non-operative management [21]. Kim et al. identified right-sided involvement, fever, and absence of rectal bleeding as factors associated with progression to surgery [23]. These adjusted analyses complement the unadjusted synthesis but do not constitute independent validation.

Across the remaining studies, adverse findings clustered within physiological deterioration, anatomical extent and severity of ischaemic injury, consistent with guideline-based severity frameworks [2,3,10]. However, these studies frequently used composite severe outcomes or postoperative endpoints and differed in predictor definitions and analytical approaches. Their findings therefore cannot be considered direct or externally validated predictors of emergency surgery. Instead, they support integrated, serial assessment and early multidisciplinary reassessment when adverse features accumulate, rather than reliance on any single variable.

A further interpretive consideration is that observed surgical treatment may reflect both disease severity and treatment-selection decisions. Physiological fitness, treatment limitations, patient preferences, clinician judgement and institutional thresholds may influence whether colectomy is offered. Patients with advanced disease may die without surgery or receive non-operative care because an operation is considered inappropriate. Consequently, the pooled estimates describe associations with observed operative treatment and cannot be assumed to exclusively indicate the need for surgery.

This review therefore complements rather than duplicates previous evidence. Sun et al. examined severe ischaemic colitis using an outcome combining surgery and death, while Qian et al. estimated the prevalence and outcomes of surgical management [7,10]. Surgery and death are related but not interchangeable endpoints, and postoperative prognosis addresses a different clinical question from progression to surgery.

The principal strengths of this review are its surgery-specific primary outcome, explicit separation of unadjusted, adjusted, composite-outcome and postoperative evidence, and study-level harmonisation of predictor definitions. The definition-restricted and Hartung–Knapp analyses were particularly informative because they revealed uncertainty that would have been obscured by presenting conventional random-effects estimates alone.

Several limitations should be acknowledged. First, only five studies contributed data to the three retained meta-analyses, with each pooled estimate based on three or four studies. Second, most cohorts were retrospective, and predictor definitions, measurement timing, reference categories, and operative terminology varied. Third, the pooled estimates were unadjusted and therefore susceptible to confounding and treatment-selection bias. Statistical heterogeneity was substantial and could not be explored reliably with so few studies. Sparse events, potential cohort overlap in non-pooled evidence, and restriction to available English-language publications introduced further uncertainty. Finally, each definition-restricted sensitivity analysis contained only two studies; the resulting Hartung–Knapp intervals were extremely imprecise. These limitations are reflected in the very low GRADE certainty for all three retained pooled predictors (Supplementary Tables S4 and S5).

Overall, the findings do not support using any individual variable as an automatic trigger for surgery. Hypotension and right-sided involvement, together with the absence of rectal bleeding, in the adjusted surgery-specific studies by Paterno et al. and Kim et al. [21,23] are better regarded as escalation signals within repeated multidisciplinary assessment and clinical judgement. Evaluation should integrate serial abdominal examination, response to resuscitation, evolving organ dysfunction, biochemical disturbance, and radiological or endoscopic evidence of advanced or irreversible injury [2,3]. Early surgical involvement is not synonymous with immediate operation; it permits explicit escalation criteria, operative planning, and critical-care preparation while response to initial management is assessed.

Future studies should prospectively enrol consecutive patients across multiple centres and apply standardised definitions of ischaemic colitis, predictor timing and emergency surgery in index admission. Surgery required at initial presentation should be distinguished from surgery following deterioration or failure of an initial conservative strategy. Studies should report crude event data alongside fully specified adjusted models and document treatment limitations, reasons for non-operative management and patient preferences. Any resulting prediction model should undergo appropriate internal and external validation, with reporting of discrimination, calibration and clinical utility before implementation in practice [40,41].

## 5. Conclusions

This systematic review and meta-analysis identifies hypotension and right-colonic involvement as clinically plausible markers associated with a higher likelihood of emergency surgery in acute ischaemic colitis. However, the evidence was derived from a few predominantly retrospective and heterogeneous studies; GRADE certainty was very low, and no pooled association was statistically conclusive using Hartung–Knapp confidence intervals. These findings do not support any single variable as an operative threshold. Their potential clinical value lies in prompting closer monitoring, early senior surgical involvement, serial multidisciplinary assessment, and timely escalation when conservative management appears to be failing, interpreted alongside response to resuscitation, evolving organ dysfunction, examination and imaging findings, patient fitness, and treatment goals.

Prospective multicentre studies using standardised predictors and outcome definitions are needed to distinguish immediate surgery from surgery after failed conservative management and to develop and externally validate clinically useful surgery-specific prediction models.

## Figures and Tables

**Table 1:** Study characteristics and summary of results.

Study (*n*)	Outcome	Reported predictors	Analysis	Timing of predictors and surgery
(A). Predictors meta-analysed and pooled				
Paterno et al., 2010 [21] (*n* = 253) USA; Yale–New Haven; 2000–2007 Retrospective	Delayed surgery: 36/241 (14.9%); 12 immediate operations excluded from analysis. IC diagnosis: Clinical signs with CT findings and/or pathological evidence.	Hypotension (SBP < 90 mmHg): OR 8.60 (95% CI 3.38–21.88). Fever (>101.5 °F/approximately 38.6 °C): OR 3.42 (95% CI 0.60–19.46).	Unadjusted odds ratios Contributes to pooled analysis	Predictors: admission vital signs. Surgery: 36 delayed operations (>12 h); 12 immediate operations (<12 h) excluded; median delay 36 h.
Kim et al., 2024 [23] (*n* = 141) South Korea; Chonnam Hwasun; 2004–2023 Retrospective	Surgery: 18/141 (12.8%). IC diagnosis: Colonoscopy or pathological evidence.	Shock: OR 4.11 (95% CI 1.09–15.41). Fever: OR 15.17 (95% CI 4.82–47.72). Right-colonic involvement: OR 3.09 (95% CI 1.07–8.90).	Unadjusted odds ratios Contributes to pooled analysis	Predictors: Shock/fever on admission (cut-offs NR); anatomy from colonoscopy or surgical biopsy. Surgery during index admission. Timing NR.
Brandt et al., 2010 [33] (*n* = 313) USA; Montefiore; 1998–2009 Retrospective	Surgery: 62/313 (19.8%). IC diagnosis: Complete visualisation at surgery or colonoscopy with histology.	Right-colon pattern: OR 6.10 (95% CI 3.35–11.09).	Unadjusted odds ratios Contributes to pooled analysis	Predictors: Anatomy from endoscopy or surgery on index admission. Surgery during index admission. Timing NR.
Medina et al., 2004 [35] (*n* = 53) Spain; Vall d’Hebron, Barcelona; 1995–2000 Retrospective	Surgery: 18/53 (34.0%). IC diagnosis: Clinical signs with CT findings or colonoscopy and pathological evidence.	Right-sided IC: OR 27.73 (95% CI 5.77–133.28).	Unadjusted odds ratios Contributes to pooled analysis	Predictors: Anatomy from endoscopy or surgery on index admission. Surgery: 12 at 24 h after admission; 6 on days 3–6.
Scharff et al., 2003 [39] (*n* = 129) USA; St Louis University Hospital; 1992–2002 Retrospective	Surgery: 76/129 (58.9%); 59 initial operations plus 17 after failed medical treatment. IC diagnosis: Clinical signs with CT findings/endoscopic and pathological evidence.	Hypotension (SBP < 90 mmHg): OR 1.27 (95% CI 0.49–3.28). Fever (>38.3 °C): OR 0.83 (95% CI 0.32–2.16). Right-sided IC: OR 1.34 (95% CI 0.60–2.96).	Unadjusted odds ratios Contributes to pooled analysis	Predictors: Hypotension/fever on admission; anatomy from colonoscopy and surgical biopsy. Surgery: 59 ≤ 12 h from admission. 17 after failed medical treatment (interval to surgery NR).
(B). Predictors quantitatively analysed but not pooled				
Paterno et al., 2010 [21] (*n* = 253) USA; Yale–New Haven; 2000–2007 Retrospective	Delayed surgery: 36/241 (14.9%); 12 immediate operations excluded from analysis. IC diagnosis: Clinical signs with CT findings and/or pathological evidence.	Peripheral vascular disease: OR 2.70 (95% CI 1.22–5.99). Chronic renal failure: OR 1.78 (95% CI 0.74–4.30).	Unadjusted odds ratios Study-level estimates without pooling	Predictors: Pre-existing comorbidities. Surgery: >12 h after admission/consultation.
Kim et al., 2024 [23] (*n* = 141) South Korea; Chonnam Hwasun; 2004–2023 Retrospective	Surgery: 18/141 (12.8%). IC diagnosis: Colonoscopy or pathological evidence.	Abdominal pain: OR 2.63 (95% CI 0.95–7.26). Rectal bleeding present: OR 0.10 (95% CI 0.02–0.47).	Unadjusted odds ratios Study-level estimates without pooling	Predictors: Baseline symptoms on admission; exact time relative to operative decision NR. Surgery during index admission. Timing NR.
Medina et al., 2004 [35] (*n* = 53) Spain; Vall d’Hebron, Barcelona; 1995–2000 Retrospective	Surgery: 18/53 (34.0%). IC diagnosis: Clinical signs with CT findings or colonoscopy and pathological evidence.	Abdominal pain: OR 1.18 (95% CI 0.38–3.70). Rectal bleeding present: OR 0.90 (95% CI 0.25–3.24). Splenic-flexure IC: OR 0.04 (95% CI 0.002–0.64). Diabetes: OR 1.86 (95% CI 0.48–7.21). Peripheral vasculopathy: OR 4.83 (95% CI 1.35–17.30).	Unadjusted odds ratios Study-level estimates without pooling	Predictors: Symptoms at presentation; pre-existing comorbidities; colonoscopy. Surgery: 12 at 24 h after admission; 6 on days 3–6. No surgical event reported for splenic flexure IC.
Scharff et al., 2003 [39] (*n* = 129) USA; St Louis University Hospital; 1992–2002 Retrospective	Surgery: 76/129 (58.9%); 59 initial operations plus 17 after failed medical treatment. IC diagnosis: Clinical signs with CT findings/endoscopic and pathological evidence.	Splenic-flexure IC: OR 0.15 (95% CI 0.05–0.50). Renal failure: OR 0.43 (95% CI 0.21–0.92). Diabetes: OR 0.26 (95% CI 0.12–0.60). Vascular disease: OR 0.12 (95% CI 0.05–0.28).	Unadjusted odds ratios Study-level estimates without pooling	Predictors: Pre-existing comorbidity history; anatomy from endoscopy or surgery. Surgery: 59 ≤ 12 h from admission; 17 after failed medical treatment, timing NR.
Study (*n*)	Outcome	Reported predictors	Analysis	Timing of predictors and outcome assessment
(C). Predictors not pooled and narratively synthesised				
(C1). Direct surgery outcomes				
Gilshtein et al., 2018 (*n* = 63) [17] Israel; Rambam, Haifa; 2011–2016 Retrospective	Surgery (12/63). IC diagnosis: Clinical signs with contrast CT in all and colonoscopy in 16.	High leukocyte count: 17.1 ± 7.2 versus 12.6 ± 6.8 × 10^9^/L (*p* = 0.03).	Unadjusted group comparison	Predictors: White cell count on admission. Surgery: Operation timing NR.
Longo et al., 1992 (*n* = 47) [19] USA; Yale–New Haven; 1978–1985 Retrospective	Surgery (31/47). IC diagnosis: Barium enema/colonoscopy or operative confirmation.	Symptom duration: 39.7 versus 16.2 h (*p* < 0.01). Coexisting medical illness: 42.9% versus 15.7% (*p* < 0.001). Hypotension: 26.3% versus 6.1% (*p* < 0.001). Ileus duration: 4.7 versus 2.1 days (*p* < 0.01).	Unadjusted group comparison	Predictors: Presenting symptoms on admission, pre-existing comorbidities; initial hypotension; ileus during treatment. Surgery: After deterioration/peritonitis; interval NR.
Paterno et al., 2010 (*n* = 253) [21] USA; Yale–New Haven; 2000–2007 Retrospective	Delayed surgery: 36/241 (14.9%); 12 immediate operations excluded from analysis. IC diagnosis: Clinical signs with CT findings and pathological evidence.	Free intraperitoneal fluid: aOR 50.00 (95% CI 6.76–333.30). Absence of rectal bleeding: aOR 14.09 (1.63–125.00).	Multivariable logistic regression	Predictors: Presenting symptoms, pre-existing comorbidity, CT during workup (exact timing NR). Predictors: admission vital signs. Surgery: 36 delayed operations (>12 h); median delay 36 h.
Kim et al., 2024 (*n* = 141) [23] South Korea; Chonnam Hwasun; 2004–2023 Retrospective	Surgery: 18/141 (12.8%). IC diagnosis: Colonoscopy or pathological evidence.	Right-sided involvement: aOR 5.92 (95% CI 1.01–34.77). Fever: aOR 24.14 (5.21–111.90). Per rectal bleeding present: aOR 0.08 (0.01–0.45).	Multivariable logistic regression	Predictors: Baseline symptoms on admission, colonoscopy; exact time relative to operative decision NR. Surgery during index admission. Timing NR.
Brandt et al., 2010 (*n* = 313) [33] USA; Montefiore; 1998–2009 Retrospective	Surgery: 62/313 (19.8%). IC diagnosis: Complete visualisation at surgery or colonoscopy with histology.	SMA-distribution disease: 34/65 (52.3%) underwent surgery versus 28/248 (11.3%) with IMA-distribution disease (*p* < 0.001). Left-colon pattern: 6/102 (5.9%) versus 56/211 (26.5%) with other patterns (*p* = 0.001).	Unadjusted anatomical comparison	Predictors: Anatomy from endoscopy or surgery. Surgery during index admission. Timing NR.
Jin et al., 2011 (*n* = 81) [34] South Korea; Chonnam National University Hospital; 2001–2010 Retrospective	Surgical-treatment group (21/81; 18 operated and 3 declined surgery). IC diagnosis: Clinical signs with CT findings or colonoscopy.	Per rectal bleeding 23.8% versus 63.3% (*p* < 0.01). Vomiting: 38.1% versus 5.9% (*p* < 0.01). Abdominal tenderness: 81.0% versus 45.1% (*p* < 0.01). Rebound tenderness: 66.7% versus 7.8% (*p* < 0.01). Heart rate ≥ 90/min: 23.8% versus 5.0% (*p* = 0.02). SBP < 100 mmHg: 33.3% versus 8.3% (*p* = 0.01). Sodium < 135 mmol/L: 38.1% versus 11.6% (*p* = 0.01). Creatinine ≥ 1.2 mg/dL: 47.6% versus 21.6% (*p* = 0.04). LDH ≥ 472 U/L: 42.8% versus 5.0% (*p* < 0.01). No factor remained significant after multivariable adjustment.	Unadjusted comparisons and multivariable regression	Predictors: Symptoms, examination findings, vital signs and laboratory tests at admission. Surgery: Operation timing NR.
Sotiriadis et al., 2007 (*n* = 273) [38] USA; Montefiore; 1998–2005 Retrospective	Surgery during admission: 61/273 (22.3%). IC diagnosis: Biopsy-proven at colonoscopy or surgery.	Isolated right-colon versus non-isolated right-colon IC: aOR 9.83 (95% CI 5.03–19.24).	Multivariable logistic regression	Predictors: Anatomy from endoscopy or surgery for all hospitalised IC patients. Surgery: During hospitalisation. Timing NR.
Scharff et al., 2003 (*n* = 129) [39] USA; St Louis University Hospital; 1992–2002 Retrospective	Surgery: 76/129 (58.9%); 59 initial operations plus 17 after failed medical treatment. IC diagnosis: Clinical signs with CT findings/endoscopic and pathological evidence.	pH < 7.34, 13/15 (87%). Total-colonic ischaemia, 13/16 (81%). Recent surgery 22/32 (69%). Inpatient onset 33/54 (61%). Predictor-specific *p* values were not reported.	Descriptive analysis	Predictors: Clinical/initial laboratory assessment, pre-existing comorbidity, anatomy from endoscopy/surgery. Surgery: 59 ≤ 12 h from admission. 17 after failed medical treatment, timing NR.
(C2). Code-defined in-hospital colectomy				
Mahfouz et al., 2022 (*n* = 106,705) [31] USA; NIS; 2016–2019 Retrospective	Colectomy during index admission: 6596/106,705 (6.2%). IC diagnosis: Principal ICD-10-CM diagnosis.	Congestive heart failure aOR 1.24 (95% CI 1.04–1.46). COPD 1.35 (1.17–1.55). Atrial fibrillation 1.39 (1.20–1.61). Coronary artery disease 1.19 (1.04–1.37). End-stage renal disease 2.19 (1.80–2.72).	Multivariable logistic regression	Predictors: Comorbidity codes. Surgery: In-hospital colectomy, timing NR.
Uhlenhopp et al., 2021 (*n* = 25,035) [32] USA; NIS; 2016 Retrospective	Colectomy during index admission in IC patients with COPD: 995/25,035 (4.0%). IC diagnosis: Principal ICD-10-CM diagnosis.	COPD aOR 1.44 (95% CI 1.01–2.06).	Multivariable logistic regression	Predictors: Comorbidity codes. Surgery: In-hospital colectomy, timing NR.
(C3). Composite severe or unfavourable outcomes and mortality				
Sadot et al., 2014 (*n* = 117) [22] USA; Mount Sinai; 2003–2008 Retrospective	Surgery/30-day death: 24/117 (20.5%). Surgery: 21/117 (17.9%). IC diagnosis: Histology from endoscopy or surgical biopsies.	Atrial fibrillation: aOR 15.51 (95% CI 1.26–190.20). Leukocytosis > 15 × 10^9^/L: aOR 5.69 (1.54–20.98). Haematocrit < 35%: aOR 4.45 (1.16–17.05).	Multivariable logistic regression	Predictors: Blood tests on admission, pre-existing atrial fibrillation. Surgery: 6 ≤ 24 h after consultation; 15 after initial failed medical treatment.
Yngvadottir et al., 2017 (*n* = 89) [24] Iceland; population-based; 2009–2013 Retrospective	Surgery/30-day death: 10/89 (11.2%). Surgery: 8/83 (9.6%). IC diagnosis: Histology from endoscopy, surgery or autopsy.	Right-colon IC: aOR 38.00 (95% CI 4.70–393.00). Simultaneous left and right colon involvement: aOR 50.00 (7.16–499.00).	Multivariable logistic regression	Predictors: Anatomy from endoscopy, surgery/autopsy. Surgery: 5 immediate; 3 delayed. Timing NR.
Fillias et al., 2022 (*n* = 205) [27] France; Lapeyronie Hospital, Montpellier; 2014–2019 Retrospective	Surgery/30-day death: 143/205 (69.8%). Surgery: 87/205 (42.4%). IC diagnosis: CT findings with endoscopic and/or surgical confirmation.	Haemodynamic instability: aOR 18.02 (95% CI 3.82–84.94). Small-bowel involvement: aOR 11.36 (2.11–61.13). Paper-thin non-enhancing colonic wall on CT aOR 38.01 (3.47–416.80) Absent stratified colonic wall enhancement on CT aOR 6.25 (1.51–25.00).	Multivariable logistic regression	Predictors: CT during acute episode, haemodynamic instability on admission. Surgery: Operation, timing NR.
Añón et al., 2006 (*n* = 85) [28] Spain; University Clinical Hospital, Valencia; 1996–2004 Retrospective	Surgery/death: 16/85 (18.8%). Surgery: 15/85 (17.6%). IC diagnosis: Clinical signs with radiologic report confirmed endoscopically, histologically or operatively.	Peritonism: aOR 48.70 (10.60–222.10). Tachycardia: aOR 7.36 (1.71–31.50). Anaemia: aOR 5.31 (1.47–19.08). Hyponatraemia: aOR 4.98 (1.47–16.80).	Multivariable logistic regression	Predictors: Clinical signs and blood tests at admission. Surgery: Operation, timing NR. Mortality window NR.
O’Neill et al., 2012 (*n* = 32) [29] UK; Queen Margaret Hospital, Dunfermline; 5-year period Retrospective	Surgery/death: 10/32 (31.3%). Surgery: 9/32 (28.3%). IC diagnosis: CT with histological confirmation following endoscopy or surgery.	Right-sided IC: 90.0% versus 18.2% (*p* = 0.0002). Abdominal guarding: 70.0% versus 9.1% (*p* = 0.001). Chronic constipation: 40.0% versus 4.5% (*p* = 0.02). PR bleeding: 20% versus 77.3% (*p* = 0.005).	Unadjusted group comparison	Predictors: Clinical signs at presentation; constipation history; anatomy from imaging/endoscopy/surgery. Surgery: 6 of 9 had surgery ≤ 24 h after admission. Mortality timing: Index admission.
Chung et al., 2010 (*n* = 153) [30] South Korea; Severance Hospital, Seoul; 2002–2008 Retrospective	Mortality: 7/153 (4.6%). Surgery: 5/153 (3.3%). IC diagnosis: Clinical and endoscopic findings.	Tachycardia ≥ 90 beats/min: aOR 4.60 (95% CI 1.41–14.70). Shock within 24 h: aOR 6.50 (2.00–21.20). Endoscopic ulceration: aOR 9.90 (2.02–48.80).	Multivariable logistic regression	Predictors: Clinical signs within 24 h of admission, ulceration on endoscopy. Surgery: Timing NR. Mortality timing: Index admission.
Montoro et al., 2011 (*n* = 364) [1] Spain; 24 centres; 2005–2007 Prospective	Surgery/death: 47/364 (12.9%). Surgery: Overall surgery-specific rate NR. IC diagnosis: Histological confirmation following endoscopy or surgery.	Abdominal pain without rectal bleeding: aOR 3.90 (95% CI 1.60–9.30). Non-bloody diarrhoea: aOR 10.00 (3.70–27.40). Peritoneal signs: aOR 7.30 (2.70–19.60). Haemoglobin < 12 g/dL: aOR 4.50 (1.80–10.70).	Multivariable logistic regression	Predictors: Clinical signs and blood tests at admission. Surgery: Timing NR. Mortality timing: Index admission.
Medina et al., 2004 (*n* = 53) [35] Spain; Vall d’Hebron, Barcelona; 1995–2000 Retrospective	Surgery or IC-related in-hospital death; 18/53 (34.0%). All 18 severe cases underwent surgery. IC diagnosis: Clinical signs with CT findings or colonoscopy and pathological evidence.	Peripheral vasculopathy: aOR 4.03 (95% CI 1.50–17.00). Right-sided IC: aOR 5.60 (1.25–19.20).	Multivariable logistic regression	Predictors: Pre-existing comorbidities and colonoscopy. Surgery: 12 at 24 h after admission; 6 on days 3–6. Mortality timing: Index admission.
Huguier et al., 2006 (*n* = 73) [36] France; Tenon Hospital, Paris; 1992–1999 Retrospective	Surgery/death: 34/73 (46.6%). Surgery: 33/73 (45.2%). IC diagnosis: Histology from surgery or colonoscopy biopsy.	Abdominal tenderness: aOR 43.40 (95% CI 3.80–49.80). Absence of lower intestinal bleeding: aOR 8.30 (2.10–33.30). Age < 80 years: aOR 9.20 (1.80–45.50).	Multivariable logistic regression	Predictors: Age and clinical signs on admission. Surgery: 13 ≤ 12 h after admission; 20 on days 2–7. Mortality timing: Index admission.
Pastor-Juan et al., 2017 (*n* = 229) [37] Spain; single centre; 1996–2007 Retrospective	Surgery/death: 45/229 (19.7%). Surgery: 22/229 (9.6%). IC diagnosis: Surgery/endoscopic biopsy.	Pancolitis: aOR 16.40 (95% CI 1.60–162.30; *p* = 0.017).	Multivariable logistic regression	Predictors: Clinical presentation and ultrasound finding. Surgery: Timing NR. Mortality timing NR.
Sotiriadis et al., 2007 (*n* = 273) [38] USA; Montefiore; 1998–2005 Retrospective	Surgery/30-day mortality: 77/273 (28.2%). IC diagnosis: Biopsy-proven at colonoscopy, surgery or autopsy.	Isolated right-colon versus non-isolated right-colon IC: aOR 6.70 (95% CI 3.57–12.58).	Multivariable logistic regression	Predictors: Anatomy from endoscopy, surgery or autopsy. Surgery: During hospitalisation, exact timing NR.
Moszkowicz et al., 2014 (*n* = 191) [20] France; single centre; 1997–2012 Retrospective	IC-related death: 84/191 (44.0%). Surgery: 174/191 (91.1%) patients underwent colonic resection. Mortality analysis included the whole mixed-treatment cohort. IC diagnosis: Endoscopic or operative diagnosis.	Age ≥ 75 years: aHR 1.75 (95% CI 1.12–2.58). Multiple organ failure: aHR 4.31 (2.67–6.95).	Multivariable logistic regression	Predictors: Age on admission, organ failure during the clinical course. Surgery: Index admission. Mortality: median 9 days from IC diagnosis (range 0–152 days).
(C4). Predictors of postoperative outcomes				
Chen et al., 2022 (*n* = 96) [18] Taiwan; China Medical University Hospital, Taichung; 2011–2020 Retrospective	Mortality after emergency colectomy: 36/96 (37.5%). IC diagnosis: Histology following emergency colectomy.	Charlson Comorbidity Index per point increase: OR 1.29 (95% CI 1.04–1.61; *p* = 0.024). APACHE II: OR 1.25 (95% CI 1.10–1.41; *p* = 0.001). Favier class 3: OR 9.02 (95% CI 1.35–60.37; *p* = 0.023).	Multivariable logistic regression	Predictors: CCI, APACHE II and Favier grading assessed from clinical presentation, comorbidities and endoscopy in index admission. Surgery: Emergency colectomy; delay defined as ≥3 days from symptom onset. Mortality timing: 30 days postoperatively or during the index admission if longer.
Beghdadi et al., 2020 (*n* = 94) [8] France; Henri Mondor, Créteil; 2008–2019 Retrospective	In-hospital or 90-day mortality after emergency open colectomy: 66/94 (70.2%). All surgical cohort. IC diagnosis: CT/colonoscopy with histology following emergency colectomy.	Lactate > 2.5 mmol/L: aOR 11.85 (95% CI 1.64–85.17). Symptoms/signs to surgery > 12 h: aOR 34.99 (7.14–171.30). Postoperative acute kidney injury: aOR 5.09 (1.13–22.80).	Multivariable logistic regression	Predictors: Preoperative lactate, delay from symptom onset, kidney injury postoperatively (exact timing NR). Surgery: 23 ≤ 12 h from onset; 71 > 12 h.
Antolovic et al., 2008 (*n* = 85) [9] Germany; Heidelberg; 2001–2004 Prospective	In-hospital postoperative mortality: 40/85 (47.1%). IC diagnosis: Preoperative radiologic studies.	ASA II–III versus IV–V: aOR 0.13 (95% CI 0.02–0.50; *p* = 0.006). Blood loss > 1000 mL: aOR 7.20 (1.90–36.80; *p* = 0.01).	Multivariable logistic regression	Predictors: ASA class, intraoperative blood loss. Surgery: 56 ≤ 24 h after admission. Timing for the remaining patients NR.
Reissfelder et al., 2011 (*n* = 177) [25] Germany; Heidelberg; 2002–2008 Prospective	In-hospital postoperative mortality for acute IC: 85/177 (48.0%). IC diagnosis: Histology following emergency surgery.	Catecholamine administration: 46/74 deaths (62.1%); *p* = 0.007. Subtotal colectomy: 58/96 (60.4%); *p* = 0.003. Acute renal failure requiring dialysis: 61/87 (70.1%); *p* = 0.0001. Low-output heart failure: 60/101 (59.4%); *p* = 0.005. Lactate >2.5 mmol/L: 46/66 (69.7%); *p* = 0.0001.	Unadjusted comparisons	Predictors: Preoperative lactate and catecholamine administration, extent of bowel ischaemia, postoperative acute renal failure, heart failure. Surgery: 71 immediate operations. Timing for the remaining patients NR.
Genstorfer et al., 2014 (*n* = 100) [26] Switzerland; University Hospital Basel; 2004–2010 Retrospective	In-hospital death: 54/100 (54.0%). IC diagnosis: Histology after colorectal resection.	Right-sided ischaemia, aOR 3.80 (95% CI 1.20–12.00). Pan-colonic ischaemia, aOR 11.00 (2.80–39.00). Lower preoperative pH, per 0.1 decrease, aOR 2.50 (1.50–4.10).	Multivariable logistic regression	Predictors: pH preoperative; anatomy at surgery/histology. Surgery: 80/100 emergency operations ≤ 6 h after decision to operate.

Values in parentheses are 95% CIs unless otherwise specified. A *p* value < 0.05 was considered statistically significant. Adjusted and unadjusted estimates are presented separately. Descriptive percentages are not odds ratios. A study may appear in more than one section because the analysis, predictor or outcome differs; there are 26 unique included studies. n refers to the full study population unless a smaller analysis cohort is specified. NIS counts are weighted admissions. All postoperative-cohort patients underwent surgery. NR means not reported. **Quantitative and narrative synthesis.** Section A presents study-level estimates for predictors included in the primary meta-analysis. Section B presents quantitative study-level estimates that were not pooled because fewer than three sufficiently comparable studies were available or predictor definitions were incompatible. Section C summarises evidence not included in the primary meta-analysis and synthesised narratively, comprising direct surgery outcomes, code-defined in-hospital colectomy, composite severe or unfavourable outcomes and mortality, and postoperative outcomes. **Cohort overlap.** Uhlenhopp’s NIS 2016 data overlap Mahfouz’s 2016–2019 data. Brandt/Sotiriadis share institution and recruitment years. Overlapping cohorts were not treated as independent evidence. **Abbreviations.** aHR, adjusted hazard ratio; aOR, adjusted odds ratio; APACHE II, Acute Physiology and Chronic Health Evaluation II; ASA, American Society of Anaesthesiologists; CCI, Charlson Comorbidity Index; CI, confidence interval; COPD, chronic obstructive pulmonary disease; CT, computed tomography; HK, Hartung–Knapp; IC, ischaemic colitis; ICD-10-CM, International Classification of Diseases, Tenth Revision, Clinical Modification; IMA/SMA, inferior/superior mesenteric artery; LDH, lactate dehydrogenase; NIS, National Inpatient Sample; OR, odds ratio; PR, per rectal; SBP, systolic blood pressure; h, hours.

**Table 2:** Confidence intervals using the Hartung–Knapp method.

Predictor	k	Primary REML Estimate (95% CI)	I^2^ (%)	τ^2^	REML with Hartung–Knapp 95% CI
Hypotension	3	3.53 (1.10–11.28)	75.0	0.7595	3.53 (0.30–41.71)
Fever	3	3.42 (0.59–19.79)	86.3	1.9681	3.42 (0.08–142.15)
Right colonic involvement	4	4.60 (1.47–14.40)	80.4	1.0863	4.60 (0.66–31.86)

Values are odds ratios (ORs) with 95% confidence intervals (CIs). All analyses used random-effects models, with between-study variance estimated using restricted maximum likelihood (REML). Hartung–Knapp adjustment retained the same study set, pooled OR and REML estimate of τ^2^, but calculated the 95% CI using a t distribution with k − 1 degrees of freedom. Hartung–Knapp CIs were used for primary inference. OR > 1 indicates greater odds of emergency surgery; OR < 1 indicates lower odds. k, number of studies; OR, odds ratio; REML, restricted maximum likelihood; τ^2^, between-study variance.

## Data Availability

All data used in this review were derived from the cited published studies. The data-extraction file and R analysis code are publicly available in Zenodo at https://doi.org/10.5281/zenodo.23659118.

## Supplementary Materials

The following supporting information can be downloaded at: https://www.mdpi.com/article/10.3390/jcm15218539/s1, Table S1: PRISMA 2020 checklist; Table S2: Full search strategy; Table S3: QUIPS risk-of-bias assessment; Table S4: Definition-restricted sensitivity analyses; Table S5: GRADE certainty of evidence for retained pooled predictors.

## Abbreviations

The following abbreviations are used in this manuscript:

CI	Confidence interval
CT	Computed tomography
GRADE	Grading of Recommendations Assessment, Development and Evaluation
HK	Hartung–Knapp
IC	Ischaemic colitis
OR	Odds ratio
PR	Per rectum
PRISMA	Preferred Reporting Items for Systematic Reviews and Meta-Analyses
PROSPERO	International Prospective Register of Systematic Reviews
QUIPS	Quality In Prognosis Studies
REML	Restricted maximum likelihood
