# Supplementary material for: Predictors of Emergency Surgery in Acute Ischaemic Colitis: A Systematic Review and Meta-Analysis
Source: J Clin Med. 2026 Nov 2;15(21):8539. doi: 10.3390/jcm15218539 (PMC13763812; doi:10.3390/jcm15218539)
Supplement: Supplementary file 1 [file jcm-15-08539-s001.zip › jcm-4644827-supplementary.pdf]

Supplementary Materials

Supplementary Table S1. PRISMA 2020 checklist

Section and topic	Item #	Checklist item	Location where item is reported
**Title**			
Title	1	Identify the report as a systematic review.	Title, p1
**Abstract**			
Abstract	2	See the PRISMA 2020 for Abstracts checklist.	Abstract, p1
**Introduction**			
Rationale	3	Describe the rationale for the review in the context of existing knowledge.	Introduction, p 1-2
Objectives	4	Provide an explicit statement of the objective(s) or question(s) the review addresses.	Introduction (final paragraph), p2
**Methods**			
Eligibility criteria	5	Specify the inclusion and exclusion criteria for the review and how studies were grouped for the syntheses.	Methods—Study selection, eligibility criteria and screening. Outcome definition and predictor harmonisation; Synthesis and statistical analysis, p2-4
Information sources	6	Specify all databases, registers, websites, organisations, reference lists and other sources searched or consulted to identify studies. Specify the date when each source was last searched or consulted.	Methods—Search strategy, p2
Search strategy	7	Present the full search strategies for all databases, registers and websites, including any filters and limits used.	Supplementary Table S2
Selection process	8	Specify the methods used to decide whether a study met the inclusion criteria of the review, including how many reviewers screened each record and each report retrieved, whether they worked independently, and if applicable, details of automation tools used in the process.	Methods—Study selection, eligibility criteria and screening, p2-3
Data collection process	9	Specify the methods used to collect data from reports, including how many reviewers collected data from each report, whether they worked independently, any processes for obtaining or confirming data from study investigators, and if applicable, details of automation tools used in the process.	Methods—Data extraction, p3
Data items	10a	List and define all outcomes for which data were sought. Specify whether all results that were compatible with each outcome domain in each study were sought (e.g. for all measures, time points, analyses), and if not, the methods used to decide which results to collect.	Methods—Outcome definition and predictor harmonisation, p3-4
Data items	10b	List and define all other variables for which data were sought (e.g. participant and intervention characteristics, funding sources). Describe any assumptions made about any missing or unclear information.	Methods—Data extraction and Outcome definition and predictor harmonisation, p3-4

Study risk of bias assessment	11	Specify the methods used to assess risk of bias in the included studies, including details of the tool(s) used, how many reviewers assessed each study and whether they worked independently, and if applicable, details of automation tools used in the process.	Methods—Study quality assessment, p4: ; Supplementary Table S3
Effect measures	12	Specify for each outcome the effect measure(s) (e.g. risk ratio, mean difference) used in the synthesis or presentation of results.	Methods—Synthesis and statistical analysis, p4
Synthesis methods	13a	Describe the processes used to decide which studies were eligible for each synthesis (e.g. tabulating the study intervention characteristics and comparing against the planned groups for each synthesis (item #5)).	Methods—Study selection, eligibility criteria and screening, p3; Outcome definition and predictor harmonisation and Synthesis and statistical analysis, p. 3–4
Synthesis methods	13b	Describe any methods required to prepare the data for presentation or synthesis, such as handling of missing summary statistics, or data conversions.	Methods—Data extraction, Outcome definition and predictor harmonisation, and Synthesis and statistical analysis, p3–4
Synthesis methods	13c	Describe any methods used to tabulate or visually display results of individual studies and syntheses.	Methods—Synthesis and statistical analysis, p. 4; Results—Table 1 and Figures 2–3, p 5–7; Supplementary Tables S3-5
Synthesis methods	13d	Describe any methods used to synthesize results and provide a rationale for the choice(s). If meta-analysis was performed, describe the model(s), method(s) to identify the presence and extent of statistical heterogeneity, and software package(s) used.	Methods—Synthesis and statistical analysis, p4
Synthesis methods	13e	Describe any methods used to explore possible causes of heterogeneity among study results (e.g. subgroup analysis, meta-regression).	Methods—Synthesis and statistical analysis, p4. Supplementary Table S5
Synthesis methods	13f	Describe any sensitivity analyses conducted to assess robustness of the synthesized results.	Methods—Synthesis and statistical analysis, p 4 Results-Sensitivity analyses, p9. Supplementary Table S5
Reporting bias assessment	14	Describe any methods used to assess risk of bias due to missing results in a synthesis (arising from reporting biases).	Not undertaken
Certainty assessment	15	Describe any methods used to assess certainty (or confidence) in the body of evidence for an outcome.	Not undertaken
**Results**			
Study selection	16a	Describe the results of the search and selection process, from the number of records identified in the search to the number of studies included in the review, ideally using a flow diagram.	Results—Study selection p 4-5, and Figure 1, p. 4
Study selection	16b	Cite studies that might appear to meet the inclusion criteria, but which were excluded, and explain why they were excluded.	Results—Study selection, p. 4 (

Study characteristics	17	Cite each included study and present its characteristics.	Results—Study characteristics, p. 4; Table 1
Risk of bias in studies	18	Present assessments of risk of bias for each included study.	Results—Study characteristics and Table 1, p5-6; Supplementary Table S3
Results of individual studies	19	For all outcomes, present, for each study: (a) summary statistics for each group (where appropriate) and (b) an effect estimate and its precision (e.g. confidence/credible interval), ideally using structured tables or plots.	Results—Predictors of surgery included in the meta-analysis, p. 5–7; Figures 2–3; Supplementary Table S4
Results of syntheses	20a	For each synthesis, briefly summarise the characteristics and risk of bias among contributing studies.	Results—Study characteristics, p5-6,; Table 1, p. 6; Supplementary Table S3
Results of syntheses	20b	Present results of all statistical syntheses conducted. If meta-analysis was done, present for each the summary estimate and its precision (e.g. confidence/credible interval) and measures of statistical heterogeneity. If comparing groups, describe the direction of the effect.	Results—Predictors of surgery included in the meta-analysis, p6-8 and Figures 2–3, p7-8
Results of syntheses	20c	Present results of all investigations of possible causes of heterogeneity among study results.	Results—Sensitivity analyses, p9. Supplementary table S5
Results of syntheses	20d	Present results of all sensitivity analyses conducted to assess the robustness of the synthesized results.	Results—Sensitivity analyses, p9, Supplementary Table S5
Reporting biases	21	Present assessments of risk of bias due to missing results (arising from reporting biases) for each synthesis assessed.	Not assessed
Certainty of evidence	22	Present assessments of certainty (or confidence) in the body of evidence for each outcome assessed.	Not undertaken
**Discussion**			
Discussion	23a	Provide a general interpretation of the results in the context of other evidence.	Discussion, p9-11
Discussion	23b	Discuss any limitations of the evidence included in the review.	Discussion p11
Discussion	23c	Discuss any limitations of the review processes used.	Discussion p11
Discussion	23d	Discuss implications of the results for practice, policy, and future research.	Discussion p11-12, Conclusion p12
**Other information**			
Registration and protocol	24a	Provide registration information for the review, including register name and registration number, or state that the review was not registered.	Methods, p2
Registration and protocol	24b	Indicate where the review protocol can be accessed, or state that a protocol was not prepared.	PROSPERO registration: CRD420261411575
Registration and protocol	24c	Describe and explain any amendments to information provided at registration or in the protocol.	Not applicable

Support	25	Describe sources of financial or non-financial support for the review, and the role of the funders or sponsors in the review.	Not applicable, p12
Competing interests	26	Declare any competing interests of review authors.	Not applicable, p13
Availability of data, code and other materials	27	Report which of the following are publicly available and where they can be found: template data collection forms; data extracted from included studies; data used for all analyses; analytic code; any other materials used in the review.	Data availability statement p13. All analyses were conducted using R version 4.6.1 and the meta package version 8.5.0.

Source: Page MJ et al. The PRISMA 2020 statement. BMJ. 2021;372:n71. doi:10.1136/bmj.n71. Reproduced under CC BY 4.0. Locations refer to the accompanying JCM-formatted manuscript; supplementary items are identified by table number.

Supplementary Table S2. Search Strategy

Database	Full search strategy	Records retrieved (n)
PubMed (All Fields)	(ischaemic colitis OR ischemic colitis OR ischaemia AND colitis OR ischemia AND colitis) AND (emergency surgery OR surgery OR urgent surgery OR colectomy OR laparotomy OR laparoscopy) AND (predict OR prediction OR risk factor OR prognostic OR prognosis)	653
Cochrane Library	(ischaemic colitis OR ischemic colitis OR ischaemia AND colitis OR ischemia AND colitis) AND (emergency surgery OR surgery OR urgent surgery OR colectomy OR laparotomy OR laparoscopy) AND (predict OR prediction OR risk factor OR prognostic OR prognosis)	25
Embase (Advanced Search)	(ischaemic colitis OR ischemic colitis OR ischaemia AND colitis OR ischemia AND colitis) AND (emergency surgery OR surgery OR urgent surgery OR colectomy OR laparotomy OR laparoscopy) AND (predict OR prediction OR risk factor OR prognostic OR prognosis)	727
Ovid MEDLINE	(ischaemic colitis OR ischemic colitis OR ischaemia AND colitis OR ischemia AND colitis) AND (emergency surgery OR surgery OR urgent surgery OR colectomy OR laparotomy OR laparoscopy) AND (predict OR prediction OR risk factor OR prognostic OR prognosis)	162
**Total records retrieved**		**1567**

Supplementary Table S3. Methodological quality assessment of the included studies using the Newcastle–Ottawa Scale

Study	Selection (maximum ★★★★)				Comparability (maximum ★★)	Outcome (maximum ★★★)			Total score
	S1	S2	S3	S4		O1	O2	O3	
Gilshtein 2018 [16]	★	★	★	–	–	★	★	★	6
Chen 2022 [17]	★	★	★	–	★★	★	★	–	7
Longo 1992 [18]	★	★	★	–	–	★	★	★	6
Moszkowicz 2014 [19]	★	★	★	★	★★	★	★	–	8
Paterno 2010 [20]	★	★	★	★	★★	★	★	–	8
Sadot 2014 [21]	★	★	★	★	★★	★	★	–	8
Beghdadi 2020 [8]	★	–	★	★	★★	★	★	–	7
Antolovic 2008 [9]	★	–	★	★	★★	★	★	–	7
Kim 2024 [22]	★	★	★	★	★★	★	★	–	8
Yngvadottir 2017 [23]	★	★	★	★	★★	★	★	–	8
Reissfelder 2011 [24]	★	–	★	★	★★	★	★	★	8
Genstorfer 2014 [25]	★	–	★	★	★★	★	★	★	8
Fillias 2022 [26]	★	★	★	★	★★	★	★	–	8
Añón 2006 [27]	★	★	★	★	★★	★	★	★	9
O'Neill 2012 [28]	★	★	★	★	–	★	★	★	7
Chung 2010 [29]	★	★	★	–	★★	★	★	–	7
Montoro 2011 [1]	★	★	★	★	★★	★	★	★	9
Mahfouz 2022 [30]	★	★	–	★	★★	★	★	–	7
Uhlenhopp 2021 [31]	★	★	–	★	★★	★	★	–	7
Brandt 2010 [32]	★	★	★	★	★★	★	★	★	9
Jin 2011 [33]	★	★	–	★	★	★	★	–	6
Medina 2004 [34]	★	★	★	★	★★	★	★	★	9
Huguier 2006 [35]	★	★	★	★	★★	★	★	–	8
Pastor-Juan 2017 [36]	★	★	–	★	★★	★	★	–	7
Sotiriadis 2007 [37]	★	★	★	★	★★	★	★	★	9
Scharff 2003 [38]	★	★	★	★	–	★	★	–	6

S1, cohort representativeness; S2, comparison-group selection; S3, ascertainment of diagnosis and predictor status; S4, absence of the outcome at predictor assessment; O1, outcome assessment; O2, adequacy of the assessment timeframe; O3, completeness of follow-up. ★, criterion satisfied (one point); –, criterion not satisfied or insufficiently reported. Comparability was awarded up to two points for control of confounding. The maximum total score was 9. Scores of 7–9, 4–6 and <4 were classified in this review as high, moderate and low methodological quality, respectively.

Supplementary Table S4. Predictors not included in the meta-analysis: findings from adjusted analyses and narrative synthesis

A. Direct surgery outcomes				
Study (n)	Outcome	Reported predictors	Analysis	Reason not pooled
Gilshtein et al 2018<br>(n=63) [16]	Operation during the index admission (12/63) versus no operation (51/63)	High leukocyte count: 17.1±7.2 versus 12.6±6.8 ×10⁹/L (p=0.03).	Unadjusted group comparison	Continuous data only
Longo et al 1992<br>(n=47) [18]	Progression to surgery after initial non-operative management (31/47) versus continued non-operative management (16/47).	Symptom duration: 39.7 versus 16.2 hours (p<0.01)<br>Coexisting medical illness: 42.9% versus 15.7% (p<0.001)<br>Hypotension: 26.3% versus 6.1% (p<0.001)<br>Ileus duration: 4.7 versus 2.1 days (p<0.01).	Unadjusted group comparison	No effect estimates or CIs
Paterno et al 2010<br>(n=253) [20]	Delayed surgery after initial non-operative management (analysis n=241)<br>12 immediate operations excluded.	Free intraperitoneal fluid: aOR 50.00 (95% CI 6.76–333.30)<br>Absence of rectal bleeding: aOR 14.09 (1.63–125.00)	Multivariable logistic regression	Adjusted results not comparable for pooling
Kim et al 2024<br>(n=141) [22]	Progression to surgery during the index admission.	Right-sided involvement: aOR 5.92 (95% CI 1.01–34.77)<br>Fever: aOR 24.14 (5.21–111.90)<br>Per rectal bleeding present: aOR 0.08 (0.01–0.45)	Multivariable logistic regression	Adjusted results not comparable for pooling
Brandt et al 2010<br>(n=313) [32]	Surgical intervention during admission (62/313). Analysed by anatomical distribution.	SMA-distribution disease: 34/65 (52.3%) underwent surgery versus 28/248 (11.3%) with IMA-distribution disease (p<0.001)<br>Left-colon pattern: 6/102 (5.9%) versus 56/211 (26.5%) with other patterns (p=0.001).	Unadjusted anatomical comparison	Different anatomical definitions
Jin et al 2011<br>(n=81) [33]	Surgical-treatment group (21/81; 18 operated and 3 declined surgery) versus medical treatment (60/81).	Per rectal bleeding 23.8% versus 63.3% (p<0.01)<br>Vomiting: 38.1% versus 5.9% (p<0.01)<br>Abdominal tenderness: 81.0% versus 45.1% (p<0.01) Rebound tenderness: 66.7% versus 7.8% (p<0.01)<br>Heart rate ≥90/min: 23.8% versus 5.0% (p=0.02)<br>SBP <100 mmHg: 33.3% versus 8.3% (p=0.01)<br>Sodium <135 mmol/L: 38.1% versus 11.6% (p=0.01)<br>Creatinine ≥1.2 mg/dL: 47.6% versus 21.6% (p=0.04)	Unadjusted comparisons and multivariable regression	Outcome included three unoperated patients.<br><br>Adjusted CI unavailable

		LDH ≥472 U/L: 42.8% versus 5.0% (p<0.01).<br>No factor remained significant after multivariable adjustment.		
Sotiriadis et al 2007 (n=273) [37]	Surgery	Isolated right-colon versus non-isolated-right-colon IC: aOR 9.83 (95% CI 5.03–19.24).	Multivariable logistic regression	Different predictor definitions
Scharff et al 2003 (n=129) [38]	Operative treatment (76/129, including 17 failed initial non-operative management) versus conservative treatment (53/129).	pH <7.34, 13/15 (87%)<br>Total-colonic ischaemia, 13/16 (81%)<br>Recent surgery 22/32 (69%)<br>Inpatient onset 33/54 (61%).<br>Predictor-specific p values were not reported.	Descriptive analysis	Descriptive data only

B. Code-defined in-hospital colectomy

Study (n)	Outcome	Reported predictors	Analysis	Reason not pooled
Mahfouz et al 2022 (n=106,705) [30]	In-hospital colectomy during the index admission in an NIS cohort of patients with IC.	Congestive heart failure aOR 1.24 (95% CI 1.04–1.46)<br>COPD 1.35 (1.17–1.55)<br>Atrial fibrillation 1.39 (1.20–1.61)<br>Coronary artery disease 1.19 (1.04–1.37)<br>End-stage renal disease 2.19 (1.80–2.72).	Multivariable logistic regression	Code-defined colectomy. Overlapping NIS population with Uhlenhopp et al 2021
Uhlenhopp et al 2021 (n=25,035) [31]	In-hospital colectomy during the index admission in IC patients with versus without COPD.	COPD aOR 1.44 (95% CI 1.01–2.06)	Multivariable logistic regression	Code-defined colectomy. Overlapping NIS population with Mahfouz et al 2022

C. Composite severe or unfavourable outcomes

Study (n)	Outcome	Reported predictors	Analysis	Reason not pooled
Sadot et al 2014 (n=117) [21]	Severe non-occlusive IC: operation or mortality.	Atrial fibrillation: aOR 15.51 (95% CI 1.26–190.20)<br>Leukocytosis >15×10^9/L: aOR 5.69 (1.54–20.98)<br>Haematocrit <35%: aOR 4.45 (1.16–17.05)	Multivariable logistic regression	Composite outcome; surgery-specific data unavailable

Yngvadottir et al 2017 (n=89) [23]	Surgery and/or 30-day mortality.	Right-colon IC: aOR 38.00 (95% CI 4.70–393.00)<br>Simultaneous left and right-colon involvement: aOR 50.00 (7.16–499.00).	Multivariable logistic regression	Composite outcome; surgery-specific data unavailable
Fillias et al 2022 (n=205) [26]	Colonic resection or death within one month of treatment initiation.	Haemodynamic instability: aOR 18.02 (95% CI 3.82–84.94)<br>Small-bowel involvement: aOR 11.36 (2.11–61.13)<br>Paper-thin non enhancing colonic wall on CT aOR 38.01 (3.47–416.80)<br>Absent stratified colonic wall enhancement on CT aOR 6.25 (1.51–25.00)	Multivariable logistic regression	Composite outcome; surgery-specific data unavailable
Anon et al 2006 (n=85) [27]	Severe IC: surgery or death.	Peritonism: aOR 48.70 (10.60–222.10)<br>Tachycardia: aOR 7.36 (1.71–31.50)<br>Anaemia: aOR 5.31 (1.47–19.08)<br>Hyponatraemia: aOR 4.98 (1.47–16.80)	Multivariable logistic regression	Composite outcome; surgery-specific data unavailable
O'Neill et al 2012 (n=32) [28]	Severe IC (surgery or death; 10/32) versus mild IC managed without surgery (22/32); nine severe cases underwent surgery.	Right-sided IC: 90.0% versus 18.2% (p=0.0002)<br>Abdominal guarding: 70.0% versus 9.1% (p=0.001)<br>Chronic constipation: 40.0% versus 4.5% (p=0.02)<br>PR bleeding: 20% versus 77.3% (p=0.005)	Unadjusted group comparison	Composite outcome; surgery-specific data unavailable
Chung et al 2010 (n=153) [29]	Severe IC: failure to improve within 2 weeks, a complication requiring surgery, or IC-related death	Tachycardia ≥90 beats/min: aOR 4.60 (95% CI 1.41–14.70)<br>Shock within 24 hours: aOR 6.50 (2.00–21.20)<br>Endoscopic ulceration: aOR 9.90 (2.02–48.80)	Multivariable logistic regression	Composite outcome; surgery-specific data unavailable
Montoro et al 2011 (n=364) [1]	Surgery and/or death	Abdominal pain without rectal bleeding: aOR 3.90 (95% CI 1.60–9.30)<br>Non-bloody diarrhoea: aOR 10.00 (3.70–27.40)<br>Peritoneal signs: aOR 7.30 (2.70–19.60)<br>Haemoglobin <12 g/dL: aOR 4.50 (1.80–10.70)	Multivariable logistic regression	Composite outcome; surgery-specific data unavailable

Medina et al 2004 (n=53) [34]	Severe IC: surgery or IC-related in-hospital death	Peripheral vasculopathy: aOR 4.03 (95% CI 1.50–17.00)<br>Right-sided IC: aOR 5.60 (1.25–19.20)	Multivariable logistic regression	Composite outcome; surgery-specific data unavailable
Huguier et al 2006 (n=73) [35]	Surgery and/or death	Abdominal tenderness: aOR 43.40 (95% CI 3.80–49.80)<br>Absence of lower intestinal bleeding: aOR 8.30 (2.10–33.30)<br>Age <80 years: aOR 9.20 (1.80–45.50)	Multivariable logistic regression	Composite outcome; surgery-specific data unavailable
Pastor-Juan et al 2017 (n=229) [36]	Severe IC: death or surgery.	Pancolitis: aOR 16.40 (95% CI 1.60–162.30; p=0.017)	Multivariable logistic regression	Composite outcome; surgery-specific data unavailable
Sotiriadis et al 2007 (n=273) [37]	Surgery and 30-day mortality.	Isolated right-colon versus non-isolated-right-colon IC: aOR 6.70 (95% CI 3.57–12.58).	Multivariable logistic regression	Composite outcome; surgery-specific data earlier reported

D. Predictors of postoperative outcomes

Study (n)	Outcome	Reported predictors	Analysis	Reason not pooled
Chen et al 2022 (n=96) [17]	Mortality after emergency colectomy for IC during the index admission.	Charlson Comorbidity Index per point increase: OR 1.29 (95% CI 1.04–1.61; p=0.024)<br>APACHE II: OR 1.25 (95% CI 1.10–1.41; p=0.001)<br>Favier class 3: OR 9.02 (95% CI 1.35–60.37; p=0.023)	Multivariable logistic regression	Postoperative cohort
Moszkowicz et al 2014 (n=191) [19]	IC-related death; 174/191 patients underwent colonic resection.	Age ≥75 years: aHR 1.75 (95% CI 1.12–2.58)<br>Multiple organ failure: aHR 4.31 (2.67–6.95)	Multivariable logistic regression	Mortality outcome
Beghdadi et al 2020 (n=94) [8]	In-hospital mortality after emergency open colectomy.	Lactate >2.5 mmol/L: aOR 11.85 (95% CI 1.64–85.17)<br>Symptoms/signs to surgery >12 hours: aOR 34.99 (7.14–171.30)<br>Postoperative acute kidney injury: aOR 5.09 (1.13–22.80)	Multivariable logistic regression	Postoperative cohort
Antolovic et al 2008 (n=85) [9]	Postoperative mortality among surgically treated patients.	ASA > III: aOR 0.13 (95% CI 0.02–0.50; p=0.006)<br>Blood loss >1,000 mL: aOR 7.20 (1.90–36.80; p=0.01)	Multivariable logistic regression	Postoperative cohort

Reissfelder et al 2011 (n=177) [24]	In-hospital mortality after surgery for acute IC; 85/177 died	Catecholamine administration: 46/74 deaths (62.1%); p=0.007<br>Subtotal colectomy: 58/96 (60.4%); p=0.003<br>Acute renal failure requiring dialysis: 61/87 (70.1%); p=0.0001<br>Low-output heart failure: 60/101 (59.4%); p=0.005<br>Lactate >2.5 mmol/L: 46/66 (69.7%); p=0.0001	Unadjusted comparisons	Postoperative cohort
Genstorfer et al 2014 (n=100) [25]	In-hospital death and major postoperative surgical complications after surgery for IC.	Right-sided ischaemia, aOR 3.80 (95% CI 1.20–12.00)<br>Pan-colonic ischaemia, aOR 11.00 (2.80–39.00)<br>Lower preoperative pH, per 0.1 decrease, aOR 2.50 (1.50–4.10)	Multivariable logistic regression	Postoperative cohort

Notes. Reported predictors findings comprise statistically significant adjusted associations and selected unadjusted or descriptive findings when adjusted estimates were unavailable. p values are retained only when no effect estimate with a confidence interval was reported. A p value <0.05 was considered statistically significant. Primary unadjusted study-level estimates included in Figures 1–2 are not repeated. Adjusted and unadjusted estimates were not combined.

Study n refers to the full study population unless the outcome cell specifies a smaller analysis cohort. Estimates are rounded to two decimal places for presentation; reported inequalities are retained.

Where a study reported multiple outcomes, the outcome most relevant to the review question is presented.

Mahfouz et al. and Uhlenhopp et al. used overlapping National Inpatient Sample data for 2016 and were therefore not combined.

Abbreviations: aHR, adjusted hazard ratio; aOR, adjusted odds ratio; APACHE II, Acute Physiology and Chronic Health Evaluation II; ASA, American Society of Anaesthesiologists; CI, confidence interval; COPD, chronic obstructive pulmonary disease; IC, ischaemic colitis; IMA, inferior mesenteric artery; LDH, lactate dehydrogenase; NIS, National Inpatient Sample; SBP, systolic blood pressure; SMA, superior mesenteric artery; CT, computed tomography; PR, per rectal.

Supplementary Table S5. Sensitivity analyses of pooled predictors of emergency surgery in acute ischaemic colitis

1. Analyses restricted to closely comparable predictor definitions

Predictor	Primary analysis	Excluded study/studies	Reason for restriction	Restricted analysis
Peripheral vascular disease	k = 3<br>OR 1.12 (0.12–10.92)<br>I² = 94.2%	Scharff et al [38]	Broader vascular disease/atherosclerosis definition.	k = 2<br>OR 3.18 (1.62–6.25)<br>I² = 0%
Hypotension	k = 3<br>OR 3.53 (1.10–11.28)<br>I² = 75.0%	Kim et al. [22]	“Shock” reported without a numerical systolic blood pressure threshold.	k = 2<br>OR 3.31 (0.51–21.58)<br>I² = 87.4%

Predictor	Primary analysis	Excluded study/studies	Reason for restriction	Restricted analysis
Fever	k = 3<br>OR 3.42 (0.59–19.79)<br>I^2 = 86.3%	Kim et al. [22]	Fever threshold not reported.	k = 2<br>OR 1.39 (0.36–5.28)<br>I^2 = 48.9%
Right-colonic involvement	k = 4<br>OR 4.60 (1.47–14.40)<br>I^2 = 80.4%	Kim et al.; Scharff et al. [22,38]	Anatomical classifications differed from the two closest definitions.	k = 2<br>OR 10.85 (2.57–45.86)<br>I^2 = 68.0%

2. Confidence intervals using the Hartung-Knapp method

Predictor	k	Primary REML estimate (95% CI)	I^2 (%)	REML with Hartung-Knapp 95% CI
Peripheral vascular disease	3	1.12 (0.12–10.92)	94.2	1.12 (0.008–164.73)
Renal failure	2	0.86 (0.22–3.43)	82.4	0.86 (1.12×10^{-4}–6,606.13)
Hypotension	3	3.53 (1.10–11.28)	75.0	3.53 (0.30–41.71)
Fever	3	3.42 (0.59–19.79)	86.3	3.42 (0.08–142.15)
Right-colonic involvement	4	4.60 (1.47–14.40)	80.4	4.60 (0.66–31.86)
Abdominal pain	2	1.84 (0.84–4.02)	5.3	1.84 (0.01–289.57)
PR bleeding	2	0.32 (0.04–2.63)	78.0	0.32 (3.57×10^{-7}–283,741.25)
Diabetes mellitus	2	0.65 (0.10–4.36)	82.8	0.65 (2.79×10^{-6}–150,783.92)
Splenic-flexure involvement	2	0.13 (0.04–0.37)	0	0.13 (1.86×10^{-4}–84.69)

Notes. Values are odds ratios (ORs) with 95% confidence intervals (CIs). OR > 1 indicates greater odds of emergency surgery; OR < 1 indicates lower odds. All analyses used random-effects models with between-study variance estimated by restricted maximum likelihood (REML). Hartung-Knapp analyses retained the primary study set, pooled OR and REML estimate of τ^2; only the CI calculation changed, using a t distribution with k − 1 degrees of freedom. Strict-definition exclusions were based on source definitions, not effect magnitude or direction. CI, confidence interval; HK, Hartung-Knapp; k, number of studies; OR odds ratio; PR, per rectum; REML, restricted maximum likelihood. Analyses were performed in R version 4.6.1 using the meta package version 8.5.0.
